# Asphyxia-Induced Bacterial Translocation in an Animal Experimental Model in Neonatal Piglets

**DOI:** 10.3390/diagnostics12123103

**Published:** 2022-12-09

**Authors:** Dimitra-Ifigeneia Matara, Rozeta Sokou, Theodoros Xanthos, Abraham Pouliakis, Antigoni Sarantaki, Theodora Boutsikou, Zoi Iliodromiti, Christos Salakos, Maria Gazouli, Nicoletta Iacovidou

**Affiliations:** 1 Neonatal Department, School of Medicine, National and Kapodistrian University of Athens, Aretaieio Hospital, 11528 Athens, Greece; 2 1st Department of Pediatrics, School of Medicine, National and Kapodistrian University of Athens, ‘Aghia Sophia’ Children’s Hospital, 11527 Athens, Greece; 3 Department of Midwifery, University of West Attica, 12243 Athens, Greece; 4 2nd Department of Pathology, School of Medicine, National and Kapodistrian University of Athens, “Attikon” University Hospital, 12462 Athens, Greece; 5 Pediatric Surgical Department, School of Medicine, National and Kapodistrian University of Athens, “Attikon” University Hospital, Medical School, 12462 Athens, Greece; 6 School of Medicine, National and Kapodistrian University of Athens, 11527 Athens, Greece

**Keywords:** neonatal asphyxia, microbial translocation, animal model, gut microbiome, intestinal ischemia, endotoxin, lipopolysacharide

## Abstract

Background: The term “bacterial translocation” (BT) refers to the migration of bacteria or their products from the gastrointestinal tract to tissues located outside it, and may occur after intestinal ischemia-reperfusion injury. The term “endotoxin” is synonymous, and is used interchangeably with the term lipopolysaccharide (LPS). LPS, a component of Gram-negative gut bacteria, is a potent microbial virulence factor, that can trigger production of pro-inflammatory mediators, causing localized and systemic inflammation. The aim of this study is to investigate if neonatal asphyxia provokes BT and an increased concentration of LPS in an animal model of asphyxia in piglets. Methods: Twenty-one (21) newborn male Landrace/Large White piglets, 1–4 days old, were randomly allocated into three groups, Control (A), Asphyxia (B) and Asphyxia-Cardiopulmonary Resuscitation (CPR) (C). All animals were instrumented, anesthetized and underwent hemodynamic monitoring. In Group A, the animals were euthanized. In Group B, the endotracheal tube was occluded to cause asphyxia leading to cardiopulmonary arrest. In Group C, the animals were resuscitated after asphyxia and further monitored for 30′. Bacterial translocation was assessed by the measurement of endotoxin in blood from the portal vein and the aorta, and also by the measurement of endotoxin in mesenteric lymph nodes (MLNs) at euthanasia. The results are given as median (IQR) with LPS concentration in EU/mL. Results: BT was observed in all groups with minimum LPS concentration in the MLN and maximum concentration in the portal vein. LPS levels in the MLNs were higher in the Group B: 6.38 EU/mL (2.69–9.34) compared to the other groups (Group A: 2.1 EU/mL (1.08–2.52), Group C: 1.66 EU/mL (1.51–2.48), *p* = 0.012). The aorta to MLNs LPS difference (%) was lower in Group B: 0.13% (0.04–1.17), compared to Group A: 5.08% (2.2–10.7), and Group C: 3.42% (1.5–5.1)) (*p* = 0.042). The same was detected for portal to MLNs LPS difference (%) which was lower in Group B: 0.94% (0.5–3) compared to Group A: 4.9% (4–15), and Group C: 3.85% (1.5–5.1)) (*p* = 0.044). Conclusions: Neonatal asphyxia can provoke ΒΤ and increased LPS concentration in blood and tissue located outside the gastrointestinal system.

## 1. Introduction

Asphyxia and hypoxia are two similar but not identical terms. They are interchangeably used to describe the lack of oxygen in an organism. Asphyxia refers to the reduced oxygen uptake due to injury or obstruction of the airways, while hypoxia describes the insufficient delivery, uptake, or utilization of oxygen by the tissues [1]. The combination of reduced oxygen supply (hypoxia) and reduced blood supply (ischemia) leads to dysfunction of all neonatal systems, such as the cardiovascular, respiratory and digestive system, and causes nerve cell death and brain damage [1,2,3,4].

The gastrointestinal tract (GIT) is particularly vulnerable to ischemic damage. Even short periods of ischemia may cause significant local tissue damage. Fetal hypoxia and perinatal asphyxia reduce bowel motility. Bacterial translocation (BT) is the migration of bacteria or their products from the GIT to tissues outside the gastrointestinal tract. Ileum is the most commonly affected part of the intestine that causes bacterial invasion, and activation of the pro-inflammatory cascade, due to the dysfunction of the intestinal barrier. One of the main possible risk factors for this clinical condition is perinatal hypoxia/asphyxia, but the pathogenesis of this phenomenon still remains unclear [5,6].

Lipopolysacharide (LPS), which originates exclusively from the cellular membrane of Gram-negative bacteria, acts as an endotoxin and has been a gold standard in the study of sepsis. The increase in plasma LPS levels disrupts the intestinal mucosal barrier and facilitates the transfer of bacteria and their components into the circulation. Intestinal barrier function is affected through different pathways, including direct effects on the repression of intestinal restitution, and indirect effects such as by promoting the release of signaling molecules from enterocytes [7,8,9]. Systemic stress causes breakdown in the intestinal mucosal barrier, leading to translocation of bacteria and endotoxin and the initiation of a signaling response within the enterocyte.

In experimental models, necrotizing enterocolitis (NEC) is characterized by circulating LPS and impaired enterocyte migration [10]. Nevertheless, data on bacteria involved in its pathogenesis is limited by the infant’s fragility, the restriction of analysis to feces and the use of culture-based methods. Perinatal asphyxia is also a complex phenomenon that affects neonatal health status. Evidence on microbial translocation, hypoxemia and tissue damage as a result of ischemia or ischemia and subsequent reperfusion, remain scarce. These parameters affect the gastrointestinal tract and take part in systemic inflammation. Due to biochemical complexities beyond the scope of studies in single-cell cultures, animal models are essential to understand these mechanisms and the effects of inflammation on the immature intestinal function [11,12]. Each animal has distinct advantages and disadvantages related to its viability, body size, genetic differences, and cost. The choice of the animal model is strongly influenced by the scientific question that the researchers seek to answer. Piglets seem to be a sufficient option in this case, so that it is possible to partially extrapolate results to the human, where communication between different cell and tissue types is complex and cannot be artificially simulated at present by a better model [13].

The aim of this study is to investigate if neonatal asphyxia provokes ΒΤ and increased concentration of LPS in blood in an animal experimental model of asphyxia in neonatal piglets.

## 2. Materials and Methods

The study protocol was approved by the Greek General Directorate of Veterinary Services (Approval number: 6304/22 December 2017) and was conducted in accordance with the Greek legislation regarding ethical and experimental procedures.

### 2.1. Animal Preparation

Study subjects were 21 male Landrace/Large-White piglets, 1–4 days old, with an average weight of 1–2.5 kg. The animals were purchased from the same breeder (Validakis registered breeder and supplier Koropi, Greece) and were transported to the research facility (Experimental Research Center, ELPEN, European Ref No. EL 09 BIO 03) at the day of each experimentation. Prior to the experimental procedure, the animals were fasted overnight but had free access to water [14]. All animals were examined by a veterinarian on the day of the experimentation and were found healthy. Animals were premedicated with intramuscular injections of 10 mg/kg ketamine hydrochloride (Imalgène, Merial Laboratorios SA, Lyon, France), 0.5 mg/kg midazolam (Dormicum, Roche, Athens, Greece), and 0.01 mg/kg atropine sulphate (Atropine sulphate, Demo, Athens, Greece), as previously described [15]. The animals were subsequently transported to the operation research facility and intravascular access through the auricular veins was obtained. Anesthesia was induced by an intravenous bolus dose of 1 mg/kg propofol (Diprivan 1% *w*/*v*; Astra Zeneca, Luton, United Kingdom) and 2 μg/kg fentanyl (Janssen Pharmaceutica, Beerse, Belgium) [16]. Whilst spontaneously breathing but anaesthetized, the animals were intubated with an endotracheal tube (Portex, 4.0 mm ID; Mallinckrodt Medical, Athlone, Ireland) and were immobilized in the dorsal recumbency on a surgical table. Additional 1 mg/kg propofol, 0.15 mg/kg cis-atracurium (Nimbex 2 mg/mL; GlaxoSmithKline, Athens, Greece), and 0.01 mg/kg fentanyl were administered to achieve synchrony with the ventilator. The mechanical ventilation was maintained with end tidal volume VT: 10–15 mL/kg, peak inspiratory pressure 19–20 cm H_2_O and respiratory rate 30–40/min, so that the end-expiratory CO_2_ was between 35 and 40 mmHg (Tonocap-TC200; DatexEngstrom, Helsinki, Finland). Self-adhesive electrodes were placed for cardiorespiratory monitoring with the animals in a dorsal recumbency. Intravenous 0.9% NaCl 10 mL/kg/h and D/W5% 5 ml/kg/h were administered in continuous infusion. Animals were kept anesthetized by infusion of propofol 8–10 mg/kg/h and cis-atracurium 0.15 mg/kg. Bolus doses of fentanyl 10 mcg/kg were administered as required by the surgical preparation and instrumentation. During the experiments the piglets were kept at a body temperature of 38 ± 1 °C, which was recorded continuously with a rectal thermometer. All animals were monitored (Mennen Medical, Envoy; Papapostolou, Athens, Greece) throughout the experiment and heart rate, electrocardiogram, and hemoglobin oxygen saturation was recorded with a pulse oximeter, with a sensor mounted on the tongue of the intubated animal. The administered oxygen concentration, during the stabilization period, was adjusted so that the SpO_2_ levels were between 90–95%. Surgical dissection with revelation of the internal jugular vein and the common carotid artery was then performed. Arterial blood gases were measured on a blood-gas analyzer (IRMA SL Blood Analysis System, part 436301; Diametrics Medical Inc, Roseville, MN, USA). For measurement of the aortic pressures, an arterial catheter (3.5 Fr, USCI CR, Bart; Papapostolou) was inserted and forwarded into the descending aorta through the right internal carotid artery. The systolic (SAP) and diastolic (DAP) arterial pressures were recorded, whereas mean arterial pressure (MAP) was determined by the electronic integration of the aortic blood pressure waveform. The right internal jugular vein was cannulated with a catheter to measure central venous pressure (CVP). The left internal jugular vein was also surgically prepared and a catheter was inserted for fluid administration. Intravascular catheters were attached to pressure transducers that were aligned to the level of the right atrium and were calibrated before their use. This allowed the recording of CVP, right atrial, and arterial pressures [17].

### 2.2. Experimental Procedure

Before the experimental procedure, the animals were randomly allocated using closed envelopes into three groups: (A) control (CON, *n* = 7), (B) asphyxia without cardiopulmonary resuscitation (CPR) performed (NoCPR, *n* = 7) and (C) asphyxia with CPR performed (CPR, *n* = 7). We used two groups to investigate the evolution of bacterial translocation during asphyxia and resuscitation and the A Group to assess whether BT occurred during anesthesia. The investigators involved in data recording, data entry, and data analysis were blinded to each animal’s allocation.

Baseline data were collected after allowing each animal to stabilize for a 30 min period. Stabilization is defined as: (a) heart rate 120–180 beats/min, (b) systolic blood pressure 70–90 mmHg, (c) average blood pressure 60–80 mmHg, (d) diastolic blood pressure 40–60 mmHg, (e) central venous pressure 2–8 mmHg, (f) hemoglobin saturation 90–95%, (g) pO_2_ 60–80 mm Hg, and (i) pCO_2_ > 35 mmHg.

In Group A, the animals were subjected only to minor aseptic procedures (instrumentation) without any other intervention. After the baseline samples of venous and arterial blood were obtained no other samples were taken again before euthanasia, which was performed 30 min after the baseline samples, with a pentobarbital overdose (200 mg/kg). The final sampling with blood and mesenteric lymph nodes (MLNs) samples was completed.

In Group B, the animals were subjected to asphyxia and not resuscitated. After collecting the baseline blood samples, the endotracheal tube was occluded to cause asphyxia defined as blood pressure <15 mmHg or heart rate <60/min whichever came first. Ventilation and administration of anesthetics were discontinued simultaneously with the onset of asphyxia. Then, the animals underwent the final (asphyxia) sampling and necropsy.

In Group C, we performed the same induction of asphyxia as in Group B, and blood samples were obtained when blood pressure was <15 mmHg or heart rate <60/min. Then, cardiopulmonary resuscitation begun immediately and blood samples were collected again. Resuscitation was performed with chest compressions and ventilation at a frequency of 3:1 ratio according to the guidelines of the European Resuscitation Council of 2021, until return to spontaneous circulation (ROSC) [18]. ROSC was defined when the values of heart rate, mean blood pressure and pulse oximetry were restored to the levels of the stabilization period with a deviation of ±10% [19]. At ROSC, a blood sample was obtained with arterial blood gases. End points of the experiment were identified as: the return of hemodynamic parameters to the values of the stabilization phase with deviation ±10% or persistent asystole after 20 min of cardiorespiratory resuscitation. When ROSC was achieved, we resumed iv propofol. After ROSC, the animals were anesthetized and monitored for 30 min, during which no further resuscitation was attempted, and then the final blood sample was taken. Adrenaline was not administered in case of failure of chest compressions (as provided by the guidelines), because it would probably act as a confounding agent to our study due to the vasoconstrictive effect of the drug, which could theoretically further aggravate intestinal damage.

Throughout the experiment, hemodynamic measurements and the duration of each period was recorded (stabilization, duration of asphyxia until hemodynamic load and duration of recovery).

**Figure 1:**
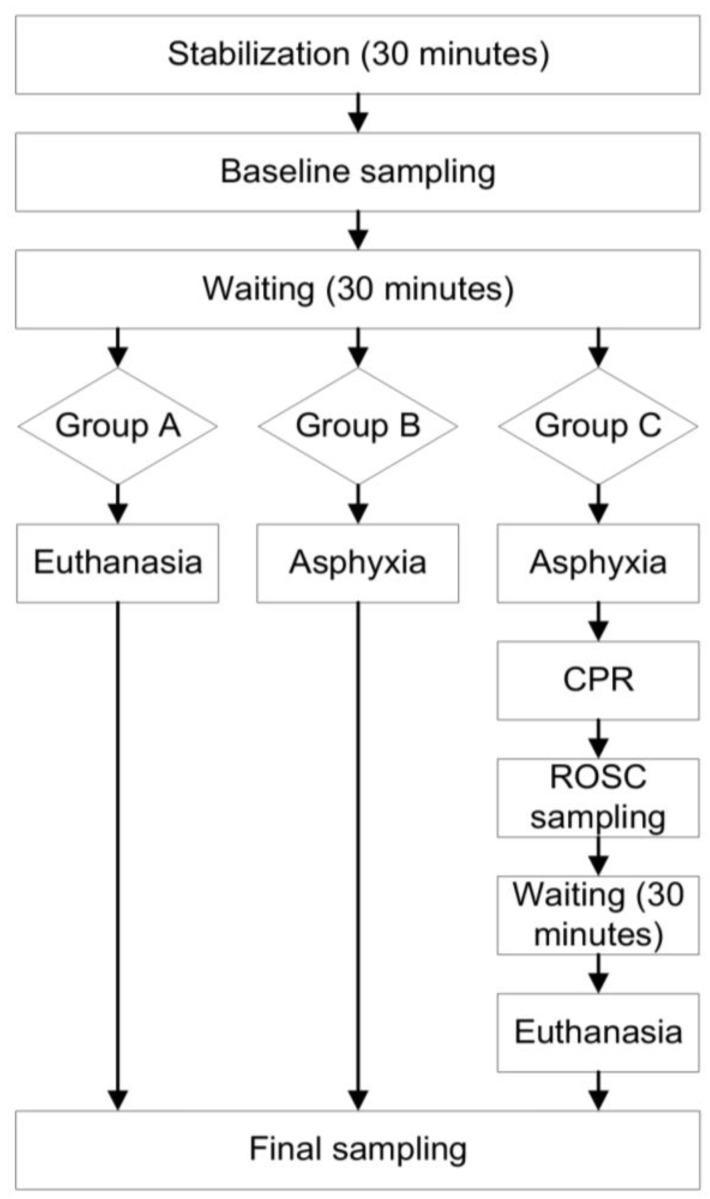
Flow chart of the study sampling and animal handling.

The animals finally were euthanized by an intravenous overdose of pentobarbital (200 mg/kg) (Dolethal, Vetoquinol SA, Lure, France). Necropsy was performed firstly under aseptic conditions, in order to measure endotoxin levels in MLNs, and also in blood from the portal vein (portal circulation) and from the aorta (systematic circulation). Figure 1 shows diagrammatically the timetable of the experiments’ sampling.

### 2.3. Experiment Outcome Points

Main outcome: presence of endotoxin in MLNs, aorta and portal vein;
Secondary outcome: hemodynamic parameters.

### 2.4. Endotoxin Analysis

Using aseptic techniques, MLNs were dissected and stored in liquid nitrogen at −70 °C until analysis. Additional samples of blood from the portal vein and the aorta were collected and immediately stored in endotoxin-free tubes (EndoGrade^®^ Glass Test Tubes, Hyglos/bioMérieux, Bernried, Germany) until analysis. Plasma levels of endotoxin in the portal and the systemic circulation and endotoxin levels from MLNs were determined using a commercially available ELISA kit (KIT LS-F15272, Life Span BioSciences, Inc., Seattle, WA, USA).

### 2.5. Statistical Analysis

During the study design, the insufficient data in literature did not allow power analysis and sample size estimation. Therefore, we were given permission to conduct a pilot study with three animals per group. The pilot data analysis revealed an adequate level of statistical significance and considering the guiding principles underpinning the humane use of animals in scientific research (three Rs), the final sample size included seven animals per group [20,21]. Arithmetic data are presented as mean ± standard deviation (SD) or using the median value and range between the 1st and 3rd quartiles (Q1–Q3 range). The normality of distributions was assessed using the Shapiro Wilk test. Categorical variables were compared between groups using the chi-square test, or Fisher’s exact test, as required. Comparisons of continuous variables between the three groups were performed using one-way ANOVA and if normality was not ensured by the Kruskal-Wallis test, furthermore pairwise comparisons were performed by the *t*-test and if normality was not ensured by the Mann-Whitney U test. Correlations between the endotoxin levels and the total time from the tracheal tube obstruction till ROSC were performed using the Spearman correlation coefficient (r_s_) since normality was not ensured for the endotoxin levels. In order to adjust for with a reference level and alleviate a possible role of individual piglets, it was also calculated the difference of the endotoxin level of the aorta from the lymph node and was extracted the percentage using as reference the lymph node level (i.e., (aorta endotoxin level—lymph node endotoxin level)/(lymph node endotoxin level)), as well as, for the portal vein from the lymph node (i.e., (portal vein endotoxin level—lymph node endotoxin level)/(lymph node endotoxin level)), these new quantities were calculated for each animal and further statistical analysis was performed on this basis. All tests were two-tailed and a value of *p* < 0.05 was considered as statistically significant. Statistical analysis was performed using the SAS for Windows 9.4 software platform (SAS Institute Inc., Cary, NC, USA).

## 3. Results

All animals in the CPR group were successfully resuscitated. There was recorded one unexpected death of an animal of Group A (control) due to technical damage of the ventilator (Control, *n* = 6). Weight characteristics of the three groups are depicted in Table 1, indicative of no statistical difference.

### 3.1. Haemodynamic Parameters & Basic Laboratory Tests

Haemodynamic and laboratory tests (total blood count and arterial blood gas) for the three groups at baseline, during asphyxia, at ROSC and at the final stage are documented in Table 2, Table 3, Table 4 and Table 5, respectively. No difference was confirmed for any of the hemodynamic parameters, for the basic laboratory data, indicative that the animals of the three groups can be considered of similar characteristics and therefore confirming that no external factor could influence the study outcomes.

Finally, significant differences were observed at the last blood gas sampling, which is physiologically expected [22] (see Table 5 for details).

### 3.2. Endotoxin Levels

**Figure 2:**
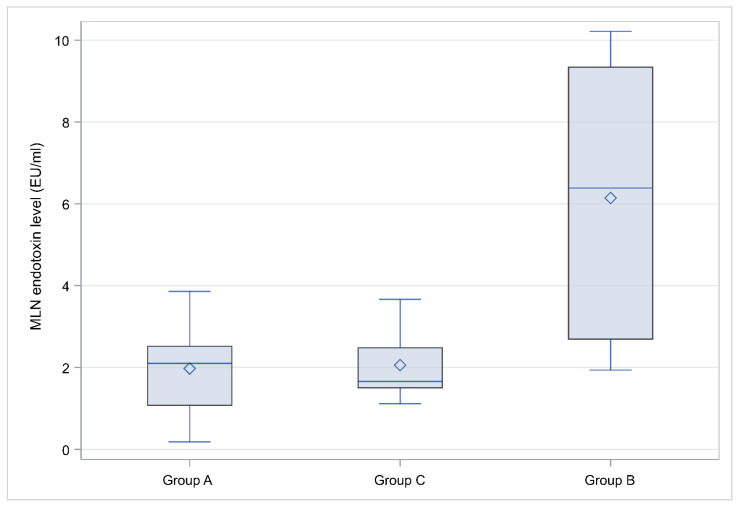
Mesenteric lymph nodes (MLN) endotoxin levels for the three animal groups. Box limits indicate the Q1 and Q3 values, the lines and diamond symbols within the boxes correspond to the median and mean value, respectively, the whisker limits correspond to the minimum and maximum value after outlier exclusion.

Endotoxin levels in the MLNs were higher in Group B: 6.38 EU/mL (2.69–9.34) compared to the other groups (Group A: 2.1 EU/mL (1.08–2.52), Group C: 1.66 EU/mL (1.51–2.48), *p* = 0,012), detailed results are presented in Table 6, along with statistical comparisons and a characteristic box and whisker plot of MLN endotoxin level for the three groups is depicted in Figure 2.

The aorta to MLNs LPS difference percentage was lower in Group B: 0.13% (0.04–1.17), compared to Group A: 5.08% (2.2–10.7), and Group C: 3.42% (1.5–5.1) (*p* = 0.042). The same trend was detected for portal to MLNs LPS difference percentage which was lower in Group B: 0.94% (0.5–3) compared to Group A: 4.9% (4–15), and Group C: 3.85% (1.5–5.1) (*p* = 0.044).

Furthermore, correlation coefficients for the aorta endotoxin levels and the time from the tracheal tube obstruction till asphyxia and total time till ROSC, were respectively r_s_ = 0.60 (*p* = 0.03), and r_s_ = 0.57 (*p* = 0.043).

## 4. Discussion

Although the scientific society has recorded significantly increasing progress in the field of resuscitation science and the pathophysiology of post-resuscitation sequel, the exact pathways of how neonatal asphyxia and post-asphyxia resuscitation affect the whole organism, remain unknown.

In this study, we tried to compare homogeneous population samples. All the piglets were male Landrace/Large-White, 1–4 days old, with a weight range from 1 to 2.5 kg. There was no significant difference in weight of the animals among the three groups and moreover the baseline hematological parameters such as blood pH and the baseline hemodynamic characteristics such as arterial blood pressure, heart rate, oxygen saturation and body temperature did not diverge. Thus, confusing factors that could influence the endotoxin level results were excluded, since the animals could be considered “similar”.

We found that LPS levels in the MLNs were significantly higher in Group B compared to the other groups. In addition, BT was observed in all groups with minimum LPS concentration in the MLNs and maximum concentration in the portal vein. The aorta to MLNs LPS difference percentage was lower in Group B compared to Group A and Group C. The same trend was detected for portal to MLNs LPS difference percentage which was lower in Group B compared to Group A and Group C.

In our study, we did not find significant differences in the LPS levels in the portal vein and the aorta, among the three groups. Considering that our animals were healthy without any concomitant disorders, we conclude that intestinal ischemia alone, as a result of neonatal asphyxia, could not increase considerably ΒΤ during that short period, indicating significant intestinal physiological reserves possibly due to preserved microcirculatory autoregulation [23]. In a previous experimental study on BT in a swine model, the researchers used 18 female Landrace/Large-White piglets, aged 10–15 weeks, and noticed BT 24 h after cardiac arrest, with significant difference in portal vein and a predominance of LPS levels in the cardiopulmonary resuscitation (CPR) group. They also examined MLNs cultures for *Escherichia coli*, with a greater increase in the cardiac arrest-CPR animals, but with no statistically significant difference. These two studies differ mainly in the animal’s gender, the animal’s age and the time of observation after ROSC till the final sampling and measurement of LPS. In addition, the pathophysiologic mechanism leading to BT differs too. In our case, the mechanism includes perinatal asphyxia, whereas in the other study, BT was related to ventricular fibrillation (VF). Our group has shown that the pathophysiology of asphyxia and VF arrest are different [24]. Moreover, in our study we used neither tissue samples, culture tests, nor markers of oxidative stress, such as malondialdehyde. We suppose that more studies in different animal models, with longer observation period and multiple time sampling would be essential for indicating the pattern of endotoxin increase during and after asphyxia and successful CPR. Probably, a combination of tissue and blood examination could give a more representative image of these complex phenomena [25].

We also found that the LPS levels in the systematic circulation (aorta) are higher in the Group C (asphyxia and cardiopulmonary resuscitation) than in the Group B (asphyxia) but not with statistical significance. LPS increases in more than 24 h after ROSC, implying that the short monitoring period of our experiments may be the reason for the non-significant differences in systemic endotoxin levels in our study. Nevertheless, we found that the longer the time to reach asphyxia, the longer time between asphyxia and ROSC. Additionally, the longer the time from the tracheal tube obstruction till asphyxia and the longer the total time till ROSC, the higher the increase in aorta LPS levels. That was an outcome consistent with other studies [26]. Another explanation for this finding could be the circulation of LPS, which either translocate through the portal venous system to the liver or enters the systemic circulation via the thoracic duct [27]. In the past, the portal vein has been considered to be the main draining system for endotoxins present in the bowel or in the peritoneal cavity. However, it is known that after superior mesenteric artery occlusion in experimental models of rabbits, systemic endotoxemia occurred in the absence of portal endotoxemia. In fact, a substantial proportion of radiolabeled endotoxins injected in the peritoneal cavity, in other studies was recovered from the thoracic duct. Additionally, after superior mesenteric artery occlusion, thoracic duct samples contained endotoxins [28].

BT may not be the only mechanism of neonatal asphyxia, post-resuscitation inflammation and sepsis. It is feasible that bacteria, macromolecules, immune cells, and cytokines are transmitted through the mesenteric lymph nodes to the systemic circulation, entering pulmonary circulation and inducing multiple organ dysfunction [29]. As analyzed above, the routes of intestinal endotoxins transport via portal venous system and via mesenteric lymph nodes can occur independently of one another, further studies are required in order to elucidate these phenomena.

Limitations to our study should be taken into consideration. Firstly, our experiments were conducted on seemingly healthy piglets, with no comorbidities and no perinatal pathological issues. This is not the case in human neonates who are confronted with asphyxia. Neonates with birth asphyxia have several risk factors involving the fetus, placenta, mother, and/or instrumentation. Secondly, the monitoring period of the Group C was limited to 30 min after ROSC. Probably, different results could be obtained in longer monitoring periods. Due to the small sample size of our study, statistical analysis may have failed to capture other factors associated with BT. Moreover, we did not use tissue samples to further examine the ischemia-reperfusion injury and the impact on BT. Another limitation of our study is that we used only male piglets and that we did not perform repeated measurements of LPS in every single animal during each experiment. However, all measurements of LPS were performed at the final sampling, i.e., for Group B, after hemodynamic burden at the point that we have defined as asphyxia and for Group C, 30 min after ROSC.

## 5. Conclusions

This is the first study presenting direct evidence regarding BT after asphyxia with resuscitation or not in a neonatal piglet model. Neonatal asphyxia can provoke bacterial translocation and increased endotoxin concentration in blood and mesenteric lymph nodes. BT was observed in all groups with minimum endotoxin concentration at the MLN and maximum concentration in the portal vein. Despite that, further scientific studies using animal models, preferably piglets, are needed, with a longer post-reperfusion monitoring period.

## Figures and Tables

**Table 1:** Characteristics of the three groups. gr = gram.

	Group A	Group B	Group C	
Characteristic	Median [Q1–Q3]	Median [Q1–Q3]	Median [Q1–Q3]	*p*
Body Weight (gr)	1600 [1400–1800]	1550 [1100–1700]	1500 [1260–1600]	0.7475

**Table 2:** Animal characteristics at baseline. Venous and arterial blood samples and hemodynamic parameters. WBC = White Blood Cells, Hb = hemoglobin, AP = Arterial Pressure, HR = Heart Rate, spO_2_ = Oxygen saturation, CVP = Central venous pressure, pH = potential of hydrogen, HCO_3_ = Bicarbonate, BE = Base Excess, Lac = Lactate.

	Group A	Group B	Group C	
Characteristic (Baseline)	Median [Q1–Q3]	Median [Q1–Q3]	Median [Q1–Q3]	*p*
WBC (10^3^/μL)	6.2 [5–10.8]	4.4 [1.4–8.4]	2.7 [2.3–5.7]	0.2607
Hb (g/dL)	6.85 [6.6–7.2]	6.7 [5.9–7.2]	6.2 [5.6–7.3]	0.9292
AP (mmHg)	55 [53–57]	58 [49–64]	53 [50–64]	0.9609
HR (beats per min)	151 [136–164]	136 [120–175]	152 [130–172]	0.9774
spO_2_ (%)	100 [99–100]	100 [99–100]	100 [99–100]	0.9646
CVP (mmHg)	3.5 [1–7]	4 [2–4]	3 [2–7]	0.9762
Temperature (°C)	38 [37.9–38.2]	38 [37.7–38.1]	38 [37.2–38.5]	0.8843
pH	7.315 [7.3–7.45]	7.3 [7.3–7.36]	7.3 [7.3–7.38]	0.7348
HCO_3_ (mEq/L)	24.8 [23.4–28.4]	26.3 [24.5–27.6]	25.3 [22.5–27.9]	0.7595
BE (mEq/L)	1.45 [−2.7–2.3]	−0.7 [−2–1.4]	−1.4 [−2.3–2.4]	0.8624
Lac (mg/dL)	0.65 [0.56–0.78]	0.65 [0.47–0.88]	0.63 [0.42–0.91]	0.8517

**Table 3:** Animal characteristics at asphyxia. NA: not applicable. Venous and arterial blood samples and hemodynamic parameters. AP = Arterial Pressure, HR = Heart Rate, pH = potential of hydrogen, HCO_3_ = Bicarbonate, BE = Base Excess, Lac = Lactate.

	Group B	Group C	
Characteristic (Asphyxia)	Median [Q1–Q3]	Median [Q1–Q3]	*p*
AP (mmHg)	27 [15–51]	28.5 [18–41]	0.7963
HR (beats per min)	57 [42–57]	57 [56–58]	0.3723
pH	6.85 [6.63–7.1]	6.75 [6.63–6.98]	0.7745
HCO_3_ (mEq/L)	23 [22.9–24]	19.1 [17.6–20]	0.1441
BE (mEq/L)	ΝA	−13.7 [−13.7–−13.7]	NA
Lac (mg/dL)	11.55 [11.5–11.6]	9.4 [5.46–12.4]	0.5637

**Table 4:** Animal characteristics at ROSC. Venous and arterial blood samples and hemodynamic parameters. AP = Arterial Pressure, HR = Heart Rate, spO_2_ = Oxygen saturation, CVP = Central venous pressure, pH = potential of hydrogen, HCO_3_ = Bicarbonate, BE = Base Excess, Lac = Lactate.

	Group C
Characteristic (ROSC)	Median [Q1–Q3]
AP (mmHg)	52 [30–90]
HR (beats per min)	158 [133–172]
spO_2_ (%)	100 [97–100]
CVP (mmHg)	4.5 [1–8]
pH	7 [6.82–7.03]
HCO3 (mEq/L)	17.2 [16.2–19.6]
BE (mEq/L)	−13.7 [−15.4–10.6]
Lac (mg/dL)	6.14 [4.83–7.03]

**Table 5:** Animal characteristics at the final stage. NA: not applicable. Venous and arterial blood samples and hemodynamic parameters. AP = Arterial Pressure, HR = Heart Rate, spO_2_ = Oxygen saturation, CVP = Central venous pressure, pH = potential of hydrogen, HCO_3_ = Bicarbonate, BE = Base Excess, Lac = Lactate.

	Group A	Group B	Group C	
Characteristic (Final)	Median [Q1–Q3]	Median [Q1–Q3]	Median [Q1–Q3]	*p*
AP (mmHg)	55.5 [53–58]	42 [42–42]	56 [50–74]	0.2696
HR (beats per min)	146 [130–162]	128 [128–128]	165 [151–181]	0.0868
spO2 (%)	100 [99–100]	99 [99–99]	100 [99–100]	0.5857
CVP (mmHg)	2.5 [2–5]	3 [3–3]	4 [2–5]	0.8041
pH	7.315 [7.3–7.49]	6.86 [6.86–6.86]	7.17 [7.1–7.2]	0.0367
HCO3 (mEq/L)	24.35 [23.4–28.3]	ΝA	22.1 [19.1–22.4]	0.0223
BE (mEq/L)	−0.85 [−2.1–1.8]	ΝA	−6 [−9–−5.1]	0.0223
Lac (mg/dL)	0.74 [0.63–0.83]	ΝA	3.37 [2.71–6.28]	0.0027

**Table 6:** Endotoxin levels at the studied locations for the three study groups. Blood samples from portal vein and the aorta.

	Group A	Group B	Group C	
Anatomic Location	Median [Q1–Q3]	Median [Q1–Q3]	Median [Q1–Q3]	*p*
Endotoxin_portal_ (EU/mL)	12.5 [11.9–14.3]	8.1 [0.7–15.7]	8.1 [2.5–13.6]	0.1917
Endotoxin_aorta_ (EU/mL)	11.7 [7.5–12.8]	7.9 [5.8–10.1]	9.3 [7.5–10.2]	0.4438

## Data Availability

The data presented in this study are available on request from the corresponding author.
